# Advances in physical diagnosis and treatment of male erectile dysfunction

**DOI:** 10.3389/fphys.2022.1096741

**Published:** 2023-01-09

**Authors:** Kun Pang, Deng Pan, Hao Xu, Yuyang Ma, Jingkai Wang, Peng Xu, Hailuo Wang, Guanghui Zang

**Affiliations:** ^1^ Department of Urology, Xuzhou Central Hospital, Xuzhou Clinical College of Xuzhou Medical University, The Affiliated Xuzhou Hospital of Medical College of Southeast University, The Affiliated Xuzhou Center Hospital of Nanjing University of Chinese Medicine, Xuzhou, Jiangsu, China; ^2^ Graduate School, Bengbu Medical College, Bengbu, Anhui, China; ^3^ Graduate School, Jiangsu University, Zhenjiang, Jiangsu Province, China

**Keywords:** erectile dysfunction, physical diagnosis, physical treatment, pathophysiological mechanisms, pharmaco penile duplex ultrasonography, dual-energy CT Arteriography, penile cavernosography, low-intensity extracorporeal shock wave therapy

## Abstract

Erectile dysfunction (ED) is the most common male sexual dysfunction by far and the prevalence is increasing year after year. As technology advances, a wide range of physical diagnosis tools and therapeutic approaches have been developed for ED. At present, typical diagnostic devices include erection basic parameter measuring instrument, erection hardness quantitative analysis system, hemodynamic testing equipment, nocturnal erection measuring instrument, nerve conduction testing equipment, *etc.* At present, the most commonly used treatment for ED is pharmacological therapy represented by phosphodiesterase five inhibitors (PDE5i). As a first-line drug in clinical, PDE5i has outstanding clinical effects, but there are still some problems that deserve the attention of researchers, such as cost issues and some side effects, like visual disturbances, indigestion, myalgia, and back pain, as well as some non-response rates. Some patients have to consider alternative treatments. Moreover, the efficacy in some angiogenic EDs (diabetes and cardiovascular disease) has not met expectations, so there is still a need to continuously develop new methods that can improve hemodynamics. While drug have now been shown to be effective in treating ED, they only control symptoms and do not restore function in most cases. The increasing prevalence of ED also makes us more motivated to find safer, more effective, and simpler treatments. The exploration of relevant mechanisms can also serve as a springboard for the development of more clinically meaningful physiotherapy approaches. Therefore, people are currently devoted to studying the effects of physical therapy and physical therapy combined with drug therapy on ED. We reviewed the diagnosis of ED and related physical therapy methods, and explored the pathogenesis of ED. In our opinion, these treatment methods could help many ED patients recover fully or partially from ED within the next few decades.

## 1 Introduction

Erectile dysfunction (ED) is defined as the inability of the penis to maintain or achieve sufficient erection hardness to satisfy satisfactory sexual performance and lasts for more than 3 months (Salonia et al., 2021), and easily be ignored by many clinical doctors. Statistic data indicates that the number of ED would rise to 322 million by 2025 globally (Ismail and El-Sakka, 2016). According to data, the prevalence of ED is 5.1% in men aged 29–30, 14.8% in men aged 40–59, 44% in men aged 60–69 (Calzo et al., 2021), and more than 50% of men over 70 are diagnosed with ED (Shamloul and Ghanem, 2013). This trend is consistent with the rise in life expectancy.

Many factors contribute to ED. Most current studies believe that ED is mainly caused by organic factors (neurogenic, vascular, diabetic, *etc.*), psychological factors (performance anxiety, stress, and mental disorders), iatrogenic factors (caused by surgical injury), and increasing age (aging) (Hellstrom et al., 2010; Chung et al., 2011; Wang, 2011; Shridharani and Brant, 2016).

At present, the clinical diagnosis methods of ED mainly include questionnaire surveys, psychological assessments, laboratory, and equipment examinations (Xiong et al., 2022). Some experts believe that clinicians should first conduct a comprehensive and targeted physical examination and questionnaire survey for patients with suspected ED (Zhang et al., 2014). The comprehensive questionnaire survey is the primary condition to help doctors diagnose ED and decide on the treatment plan. The most widely used questionnaire to evaluate male sexual function is the International Index of Erectile Function (IIEF)-5 (Vickers et al., 2020).

Currently, the treatment methods for ED mainly include lifestyle adjustment, psychotherapy, drug therapy, physical therapy, and surgical therapy (Liu et al., 2020a). However, the effect of lifestyle adjustment therapy is not obvious in the treatment of ED, and there is a lack of interventional studies (Yafi et al., 2016). The high cost of counseling and the uncertainty of efficacy of psychotherapy pose difficulties for most patients (Emanu et al., 2016). Because each person’s anxiety factors are different, there are no standardized protocols for psychosomatic pharmacological treatment of ED (Melnik et al., 2011). The efficacy of drug therapy is currently positive, but some patients do not respond to the drug, such as patients with severe vascular ED, diabetic ED or neurogenic ED. Besides, some adverse effects also limit the application of drug therapy in ED patients (Brock et al., 2002). Few patients choose surgical therapy because of the high cost and risks. Taking prosthetic implants for example, it costs more than $20,000 and have a high risk of infection (Stephenson et al., 2005). Therefore, physical therapy has become the choice of more and more ED patients because the relatively certain efficacy and the acceptable cost. Given the increasing prevalence and low overall diagnosis rate of ED, we review the pathophysiological mechanism as well as the benefits and drawbacks of standard clinical diagnostic equipment and physical therapy device for ED in order to give physicians a better systematic understanding of the diagnosis and physical treatment of ED.

## 2 Pathophysiological mechanism

### 2.1 Organic ED

#### 2.1.1 Neurogenic ED

The central regulation of erectile involves various primary afferents, spinal interneurons, sympathetic nerves, parasympathetic nerves, and so on (Agochukwu-Mmonu et al., 2020). Neurological disorders may lead to abnormalities in the endocrine system or the cardiovascular system, which can affect sexual function (Agochukwu-Mmonu et al., 2021). Approximately 10%–19% of the etiology of ED can be classified as neuropathy, which may be central, peripheral, or both (Del Popolo et al., 2020). The erection process requires stimulation of the hypothalamus received by the tactile, visual, and auditory sense organs followed by signals transmitted by neurons. This process may require an entire neural pathway (autonomic nervous system), so damage to any point in that neural pathway that disrupts the transmission of signals may result in ED (Giuliano, 2011). The study of Hicks et al. (Hicks et al., 2021) proved that peripheral neuropathy affected male erectile function to some extent, and they mentioned the highly overlapping relationship among peripheral neuropathy, ED, and cardiovascular diseases, which proved that peripheral neuropathy was a new risk factor of ED. Neuropathy can also be caused by being overweight, studies have shown that compared with normal weight, obesity in peripheral neuropathy (especially small nerve fibers lesions) is the more common form, Herman et al. (Herman et al., 2007) confirmed that corneal nerve fiber density and length are associated with the diagnosis of ED, but has no obvious relation with the severity of ED. Neurological injury due to trauma can also cause ED, depending on the degree of neurological injury and the integrity of the nerve. Trauma can cause damage to the cavernous nerve, the axonal density and conduction velocity of this nerve will be reduced, which leads to the occurrence of ED (Yin et al., 2013). Sympathetic and parasympathetic nerves are involved in the erection process of the penis, emanating from the lumbar spine and sacral root. Damage to these nerves blocks the conduction of corresponding nerve signals, directly leading to ED (Giglia and Stein, 2019). ED is involved in many surgical complications in clinical practice. For example, one of the most common complications after radical prostatectomy is ED, which is usually caused by intraoperative injury of the cavernous nerve (Lima et al., 2021). This nerve injury induces protein 1, Ninjurin-1, to participate in the neuroinflammatory response, which resulted in ED (Yin et al., 2013). Long-term postoperative complications of colorectal surgery due to autonomic nerve injury in the pelvis also include ED (Giglia and Stein, 2019). In a prospective study of 50 subjects by Hande Gokce, the incidence of ED after rectal cancer surgery was found to be about 10%–35%. The etiology of these ED patients is considered to be related to vascular nerve damage during the rectal surgery. (Gokce and Ozkan, 2019).

#### 2.1.2 Diabetic ED

**FIGURE 1:**
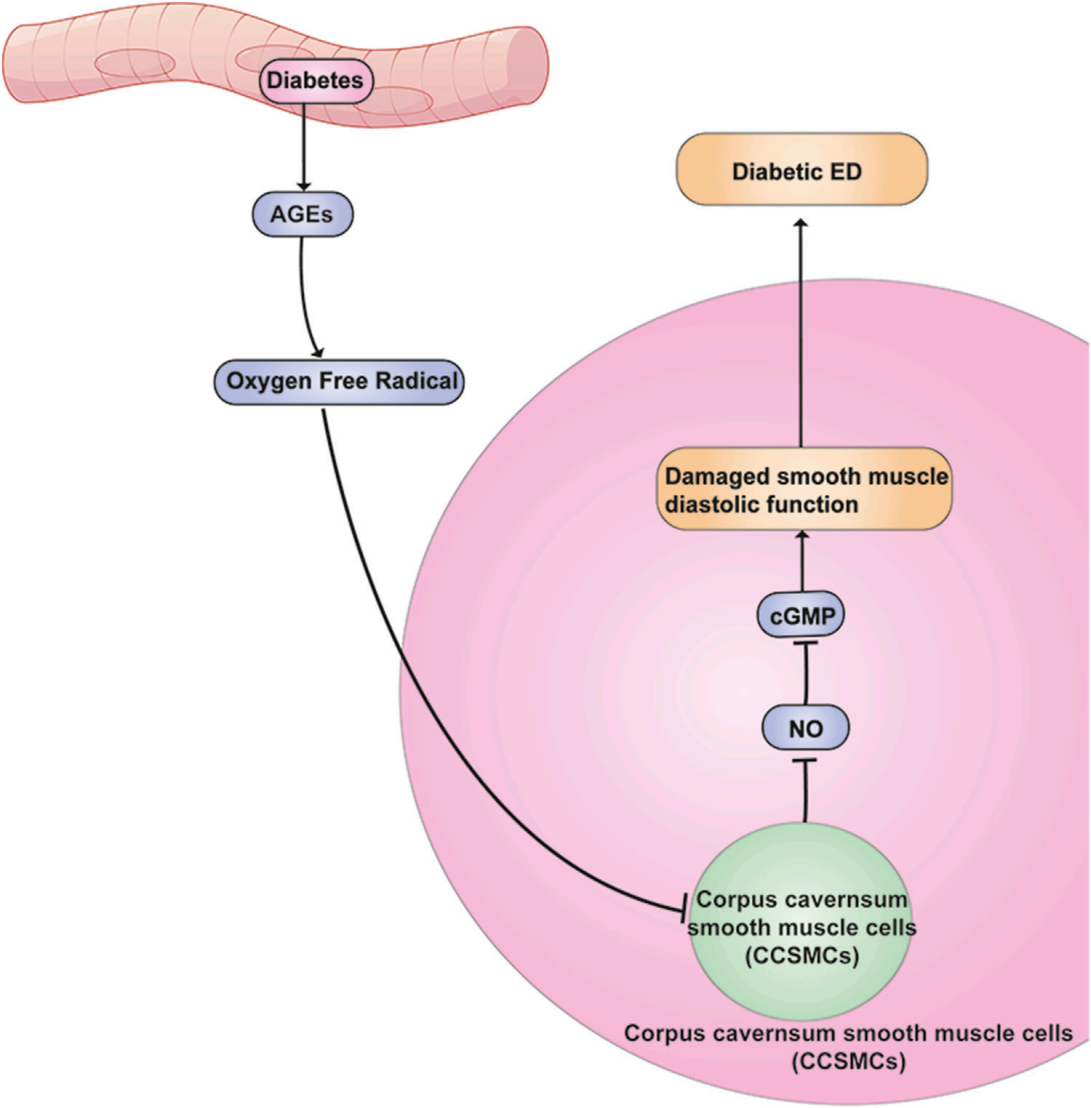
Mechanism of chronic hyperglycemia leading to ED. Elevated blood glucose leads to elevated AGEs, and their elevation leads to damage to cavernous smooth muscle cells affecting the diastolic function of this smooth muscle, which in turn leads to ED.

Diabetes mellitus is a metabolic disease with hyperglycemia caused by defective insulin secretion, defective insulin action, or both (Cloete, 2022) (Figure 1). Persistently high blood glucose levels can lead to nerve and blood vessel damage, cardio-cerebral circulatory complications, and even death (Faselis et al., 2020). Diabetes is considered as a major risk factor for ED, and the association between diabetes and the development of ED has been documented in animal models and humans since 1970 (Gur et al., 2014). Chronic hyperglycemia may lead to impaired nitric oxide (NO) synthesis and cycloguanosine monophosphate (cGMP) pathway, increased reactive free radical level, upregulated of RhoA/Rho kinase pathway, and damaged nerve function, which may be the mechanisms of ED in diabetic patients (Gurbuz et al., 2022). The persistent state of hyperglycemia will lead to an increase in advanced glycation end products (AGEs), which are the final products of amino non-enzymatic glycation of proteins, lipids, and nucleic acids in human tissues. Increased expression of AGEs in the corpus cavernosum of diabetic patients may lead to changes in tissue structure, such as thickened vascular walls, decreased elasticity, endothelial dysfunction, and atherosclerosis. This process can produce overloaded peroxynitrite, which can lead to oxidative damage to a number of important biomolecules, resulting in ED (Trebaticky et al., 2019). The cGMP mainly induces the relaxation of cavernosal vascular smooth muscle through cGMP dependent protein kinase-1 (PKG-1) changing the levels of intracellular and extracellular calcium and potassium ions. Some researchers believe that the occurrence of ED in patients with diabetes is related to the reduction of cGMP and the impaired relaxation of cavernosal smooth muscle due to the oxygen free radicals produced by AGEs induced related cellular oxidative damage and the quenching of NO (Thorve et al., 2011). Nerve damage caused by chronic hyperglycemia can affect different sensory patterns as well as autonomic function (Sharma et al., 2020). Some sensory diagnostic devices can be used to evaluate sensory functions such as vibration perception, pressure perception, and heat perception, so as to improve diagnostic sensitivity in clinical practice (Freeman, 2014). Morning testosterone levels can also be used as an auxiliary diagnosis method of diabetic ED. Diabetes-associated ED patients often have low morning testosterone level (Gianatti and Grossmann, 2020). However, diabetes is not the unique etiology of low testosterone levels, therefore, it is critical to identify other diseases that can affect testosterone levels, such as endocrine disorders and urinary system diseases. (Onyeji and Clavijo, 2022).

#### 2.1.3 Vasogenic ED

ED can be caused by a variety of vascular factors, such as atherosclerosis, arterial injury, and stenosis, penile venous fistula (Hoppe and Diehm, 2020; Sayadi et al., 2021; Wang et al., 2021). The role of monocyte/macrophage accumulation in vascular disease is not negligible (Davis and Gallagher, 2019; Miyata et al., 2021). Macrophages can stimulate plaque formation in blood vessels and play an important role in vascular injury (Marchio et al., 2019). Abnormal lipid metabolism and monocyte/macrophage interactions can also accelerate the formation of atherosclerotic plaques, which is closely related to the development of ED (Randrup et al., 2015). It has been shown that macrophages can affect endothelial function through macrophage-derived myeloperoxidase (MPO) - dependent ox-LDL (Mox-LDL) (Boudjeltia et al., 2013). MPO can promote impaired endothelial function and intravascular plaque instability, and Mox-LDL can stimulate macrophages to produce reactive oxygen species (ROS) and secrete cytokines to affect endothelial function (Calay et al., 2010). Endothelial damage may alter the state of blood flow within them, which in turn leads to ED (Salvio et al., 2021). Miner et al. (Miner et al., 2012) showed that vasogenic ED preceded coronary heart disease in younger ED patients. The pathology of arterial ED is atherosclerosis of the internal pudendal artery, which prevents the corpus cavernosa from receiving sufficient blood flow to achieve erectile status (Pereira et al., 2013). Studies have shown that as vascular smooth muscle cells proliferate, collagen and fibrosis increase, leading to the thickening of the vessel wall and narrowing of the lumen. This made a decrease in blood flow to the penis, and resulted in ED (Hannan et al., 2011). The main diagnostic methods of vascular ED include color dual Doppler ultrasound, selective penile angiography, magnetic resonance imaging, and intravascular injection of vasoactive drugs (Ma et al., 2020).

### 2.2 Psychogenic ED

Psychological problems such as anxiety, stress, and mental disorders can significantly affect the development of ED (Tan et al., 2012), there is a higher risk of ED in people with psychosis who are at very high risk (Bourdeau et al., 2012). Various psychotropic drugs are widely used in young adults with psychosis-related ED. Some studies have shown that some antipsychotic drugs can affect the dopamine D2 receptor pathway, which in turn affects erectile function. (Reichenpfader et al., 2014; Chen et al., 2019). The study of Macdonald et al. verified that psychological problems such as low self-esteem, emotional retardation, and sleep disorders not only directly affected the sexual function of patients, but also showed that the severity of mental problems was positively correlated with the severity of ED (Macdonald et al., 2003). People who experience problems with erectile function are more likely to develop anxiety, which feeds back on them over time, leading to ED.

## 3 Physical diagnosis of ED

The incidence of ED is increasing over years, (Matz et al., 2019). Aging, smoking, and unhealthy lifestyle are all risk factors for ED. The diagnosis of ED is still in the process of being improved, for the purpose to achieve early detection and diagnosis, in order to draw up better treatment plans for ED patients. With the increasing demand for quality of life, sexuality must also be considered.

### 3.1 Basic parameters of penile erection

The basic parameters of penile erection, including the length and circumference of the penis and the temperature of the head during erection, can be easily obtained by a ruler. These parameters can be used as a diagnostic method for those who were suspected with ED, but should not be used alone as evidence for the diagnosis of ED.

### 3.2 Nocturnal penile tumescence (NPT)

NPT times are one of the reliable methods to distinguish psychological ED from organic ED. In clinical practice, many methods can be used to measure NPT, such as sleep laboratory testing, stamp tests, nocturnal electrobioimpedance volumetric assessment, and the Mercury strain (Zou et al., 2019). However, these methods have many obvious drawbacks such as being time-consuming, the inability to objectively analyze the causes and only arrive at yes and no results, and the possibility of other reasons affecting the test results during the test (Qin et al., 2018a). Until 1985, Bradley et al. invented Rigiscan, a portable device that measures penile circumference, axial and circumferential dilation rates, penile erection frequency, duration, and penile stiffness (Bradley et al., 1985). Zhang et al. found that the erection duration obtained by Rigiscan can be used to distinguish between arterial and venous ED, with a sensitivity of 81.4% and a specificity of 100% at the cutoff value of 12.5 min while predicting venous ED (Zhang et al., 2020). However, whether NPT times measured by Rigiscan are reliable in distinguishing psychological from organic ED remains debatable (Jannini et al., 2009). Some researchers believe that this method has many influencing factors, difficult to repeat, and increases the economic burden. Moreover, it can only make a simple distinction, and cannot identify the etiology of ED (Liu et al., 2020b). Wang et al. found that oral Phosphodiesterase inhibitor-5 (PDEi-5) concomitant with audiovisual stimulation, using Rigiscan to objectively assess penile swelling and tonicity, was a better way to differentiate between psychogenic and organic ED (Wang et al., 2018a).

### 3.3 Pharmaco penile duplex ultrasonography (PPDU)

Ultrasound assessment of ED was introduced in 1985 by Lue et al. (Lue et al., 1985), PPDU refers to color Doppler ultrasound combined with injection of intravascular active substances in the corpus cavernosum. The most used vasoactive agents are papaverine alone or a combination of papaverine, phentolamine, and prostaglandin 1 (PGE1) (Mutnuru et al., 2017). The advantage of PPDU is that it enables objective, minimally invasive assessment of hemodynamics at a relatively low cost (Golijanin et al., 2007). Blood flow to the penis can be assessed by color Doppler ultrasound, which can accurately assess venous leakage. Peak systolic velocity (PSV), end-diastolic velocity (EDV), and electrical resistance index (RI) are commonly used to evaluate arterial blood flow by ultrasonography (Jung et al., 2018). According to the standard operating procedures published by the International Society of Sexual Medicine in 2013, PSV is an accurate predictor of arterial disease in ED patients: PSV >30 cm/s and EDV<3 cm/s indicate normal arterial supply, while PSV<25 cm/s indicates arterial insufficiency. Venous occlusive dysfunction was defined as PSV >30 cm/s, EDV >6 cm/s, and RI <.6 (Sikka et al., 2013). Although PPDU can be used to identify arteriovenous ED, it has some limitations. It is complex, expensive, and censor-dependent; more importantly, the examination requires complete relaxation of the smooth muscle in order to truly reflect the vascular condition, and requires a relatively high volume of drug injection. Patients will inevitably experience tension and anxiety during the examination, which will lead to large errors in the examination results (Ma et al., 2020). It is these limitations that contribute to the high false positive rate of this inspection method (Caretta et al., 2019).

### 3.4 Selective internal pudendal arteriography (IPA) by digital subtraction angiography (DSA)

Selective IPA is a reliable and effective method for the diagnosis of arterial ED because it can show the morphological characteristics of the terminal branches of the internal pudendal artery (Wang et al., 2019a). It can also help us to locate arterial lesions and assess the arterial blood supply by DSA technology (Wang et al., 2019a). However, this examination is an invasive examination, which may cause bleeding at the puncture site, pain to patients, and even arterial perforation, *etc.* Moreover, the addition of DSA technology will prolong the examination time and increase the patient’s cost, making this technique rarely used in clinical practice.

### 3.5 Dual-energy CT arteriography (D-e CTA)

In recent years, dual-energy CT angiography has been more and more commonly used in the diagnosis and monitoring of male diseases (Rajiah, 2020). Dual-energy CT angiography is a novel, non-invasive and effective method to evaluate the penile arterial system, and shows high sensitivity and specificity in the diagnosis of arterial ED (Wang et al., 2022a). In 2001, Kalwanishi et al. used CTA and multi-slice CT for the diagnosis of ED and compared with DSA, proving their high accuracy and superior to DSA in the assessment of internal artery stenosis (Kawanishi et al., 2001). In addition, compared with DSA, CTA is relatively less invasive and cheaper. With more advanced imaging technology in the future, CTA can better surpass and replace DSA in the diagnosis of arterial ED.

### 3.6 Penile cavernosography

Penile cavernosography has been used to explore the venous system of the penile corpus cavernosum since the 1980s (Hsu et al., 2015). Up to now, penile cavernosography has been widely used to evaluate venous occlusive dysfunction in ED organic ED patients, and it has become the gold standard for the diagnosis of venous ED (Glina and Ghanem, 2013). Studies have shown that drug-induced cavernosography alone may lead to insufficient priapism, which is misdiagnosed as venous leakage. Dynamic continuous perfusion must be used to induce complete priapism in order to obtain more realistic detection results (Song et al., 2015). However, due to the influence of other veins, bones, or cavernous shadows, cavernosography cannot accurately display the target vein and accurately assess the leakage site (Xu et al., 2017a). Furthermore, because cavernosography is more invasive than PPDU, it is not recommended in some cases at which PPDU is sufficient to diagnose venous ED unless surgery or venous embolization is required. (Soylu et al., 2021).

### 3.7 320-Detector row dynamic volume CT (4D-CTA)

The 320-detector dynamic volume CT is composed of 320 detectors with a thickness of about .5 m and a width of about 16 cm along the *Z*-axis, and the gantry rotation time is 350 m (Hoe and Toh, 2009). Compared with DSA, 4D-CTA is less expensive and less time-consuming, involves no invasive procedures that could result in problems like thrombosis. (Gang et al., 2012; Biswas et al., 2015). 4D-CTA can instentlydisplay the flow of contrast agent in blood vessels following contrast agent injection t, and it can perform continuous volume scanning within a set time, which is used to estimate various conditions of hemodynamics and vessel morphology (Han et al., 2012; Biswas et al., 2015). Previously, 4D-CTA was frequently employed to identify myocardial ischemia and developing venous anomalies brought on by atherosclerotic heart disease (Dewey et al., 2009). In an article by Xu et al., they discovered that 4D-CTA was equally as accurate as CDDU in diagnosing arterial ED, with a specificity of 93.9% and an accuracy of 87.7%. (Xu et al., 2017b).

### 3.8 Discussion

The causes of ED are diverse, including arterial, venous, neurogenic, psychogenic, and medically induced injuries, which requires the availability of screening tools for each cause. Invasive tests such as PPDU and Penile cavernosography, even if they are less traumatic to the patient, still add to the patient’s psychological burden and naturally have an impact on the diagnosis; other non-invasive tests are expensive and add to the patient’s financial burden. For the above-mentioned examination methods, the author prefers dual-energy CT. First, it is a non-invasive examination method. This ensures that the patient can face the examination with a relatively calm state of mind and will not have much influence on the accuracy of the examination results. Secondly, dual-energy CT takes less time, shortens the patient’s hospitalization period, and increases the patient’s medical compliance. Finally, the accuracy and sensitivity of this test are high, which can accurately analyze the patient’s lesion site and give clinicians more precise guidance on the direction of treatment. Therefore, we believe that the future development direction of diagnostic technology needs to March in the direction of convenient, affordable, and non-invasive examination. With the development of image technology, CT imaging technology will be greatly improved, and CT examination is relatively convenient, inexpensive, and non-invasive, which should become the mainstream choice for diagnosing ED in the future.

## 4 Physical therapy of ED

Currently, physical methods commonly used to treat ED include Vacuum erectile devices (VED), low-frequency electrical stimulation, low-intensity extracorporeal shock waves, Chinese acupuncture, and other treatment methods.

### 4.1 VED

**FIGURE 2:**
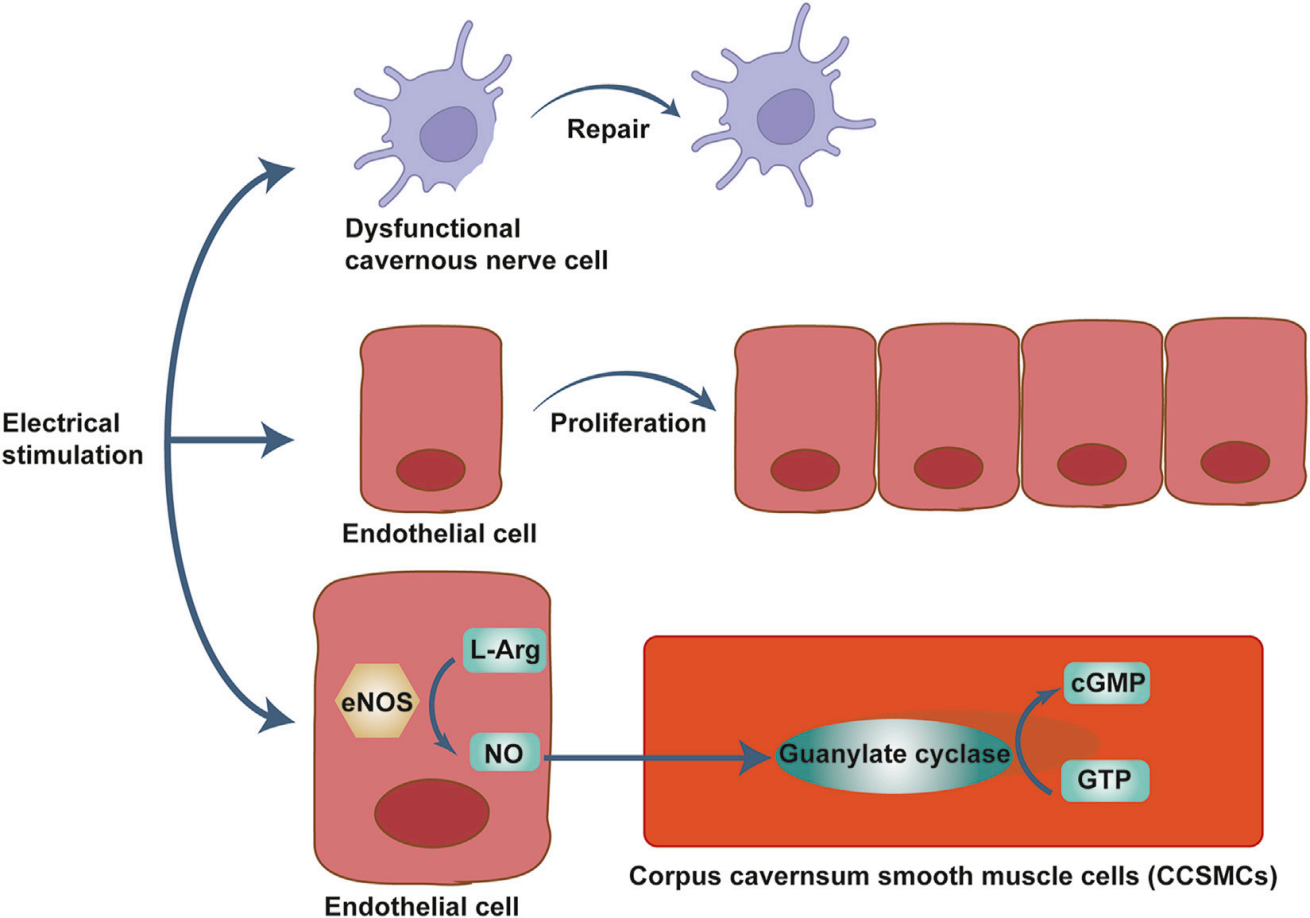
Mechanism of ES for the treatment of ED. Electrical stimulation treats erectile dysfunction by promoting cavernous smooth muscle proliferation, repairing cavernous nerves, and increasing endothelial cell NO.

Most VED consist of a shrink ring, a cylinder and a pump powered manually or by battery power (Beaudreau et al., 2021). It employs negative pressure to dilate the cavernous venous sinus, increase the perfusion of cavernous artery and venous blood, and ultimately achieve the goal of producing penile erection. An external constriction ring is placed at the base of the penis to prevent blood flow during intercourse in order to maintain an erection, but the ring should not be placed for more than half an hour (Yuan et al., 2010; Lin and Wang, 2013). It is used to promote the recovery of penis function and maintain penis length (Ma et al., 2021). Animal experiment by Ma et al. had demonstrated that the erectile response induced by the vacuum erection device may increase the smooth muscle/collagen ratio by decreasing hypoxia-inducible factor-1 and transforming growth factor-1, thereby improving penile blood flow (Yuan et al., 2010; Brison et al., 2013). In addition, the device can only increase the oxygenation of the corpus cavernosum without the use of a shrink ring. This method can bypass the limitations of oral drugs and directly achieve an artificial erection, but it requires a normal and intact cavernous nerve to produce an erection (Lin and Wang, 2013; Beaudreau et al., 2021). Compared to other penile rehabilitation therapies, VED therapy has the advantages of being non-invasive and having fewer systemic side effects (Brison et al., 2013; Sultana et al., 2022) (Figure 2).

The study by Sherry A Beaudreau et al. found that the correct use of the device resulted in an erection to complete normal intercourse in 90.7% of patients (49 of 54 patients), all of whom indicated that they would recommend the device to other ED patients. Approximately 93.9% of patients reported that their quality of sex life was satisfactory or very satisfactory after treatment with the VED (Beaudreau et al., 2021). In a study by Khayyamfar et al., the erectile success rate of the subjects reached 100%, and it is verified that VED is not affected by the etiology of ED (diabetic, venous occlusion dysfunction, arterial origin, *etc.*) in achieving erection (Khayyamfar et al., 2014). Although most patients and their partners are satisfied with the device, studies had shown that the use of the device causes a lot of discomfort for some patients. For example, insufficient lubrication may lead to bruising of the penis, numbness and/or pain of the penis, cold sensation of the penis and inability to ejaculate (Brison et al., 2013; Lin and Wang, 2013; Beaudreau et al., 2021). About 25% of the patients in a study reported some physical discomfort during and after use of the device (Beaudreau et al., 2021). According to some studies, the negative pressure suction device also brings some psychological discomfort to patients, such as frustration and lack of autonomy (Brison et al., 2013; Lin and Wang, 2013; Beaudreau et al., 2021; Ma et al., 2021; Sultana et al., 2022).

VED has become a common method of postoperative penile rehabilitation (Lima et al., 2021). After radical prostatectomy, at least 85.8% of ED patients received penile rehabilitation, including VED (Tal et al., 2011; Lima et al., 2021; Zhang et al., 2022). Studies have demonstrated that ligation of the internal paraarteries of the genitals during radical prostatectomy may lead to nerve damage and decrease arterial inflow, which is a potential cause of ED after radical prostatectomy (Lin and Wang, 2013; Zhang et al., 2022). In terms of efficacy, Dalkin et al. conducted a study of 42 patients who had undergone nerve-preserving radical prostatectomy and discovered that only one of 36 patients who received VED had a penis length reduction more than 1 cm. Regular use of VED in the early postoperative period has been verified to be beneficial for the preservation of penile length (Karakus and Burnett, 2020). The Pajoovic et al. (Pajovic et al., 2017)study included 50 patients with type I and type II diabetes ED, treated with VED and found that 85% of patients had positive returns. Therefore, VED therapy can be used as an alternative to pharmacotherapyz (Dalkin and Christopher, 2007; Karakus and Burnett, 2020). Some studies have shown that VEDs can also be used in combination with laser illumination and are more effective than either alone (Moskvin and Ivanchenko, 2014).

VEDs are non-invasive and very effective in treating ED and improving sexual partner relationships, with a high success rate and very few side effects (Liu et al., 2017), particularly in patients with ED after radical prostatectomy. In ED patients after radical prostatectomy, early treatment with VED can significantly improve erectile function (Qin et al., 2018b). Common adverse effects of VEDs include penile contusion due to improper use, especially in patients who are taking or have recently taken anticoagulants, penile numbness and/or pain, penile coldness, and ejaculation disorders (Fode et al., 2020). The device may also cause some psychological discomfort to the patient, such as frustration and feelings of lack of autonomy. Therefore, combining negative pressure suction device treatment with psychotherapy will be more effective. Moreover, the optimal duration of VED treatment for ED, the oxygen saturation in the cavernous body during treatment, and other factors need to be further studied.

### 4.2 Electrical stimulation (ES)

**FIGURE 3:**
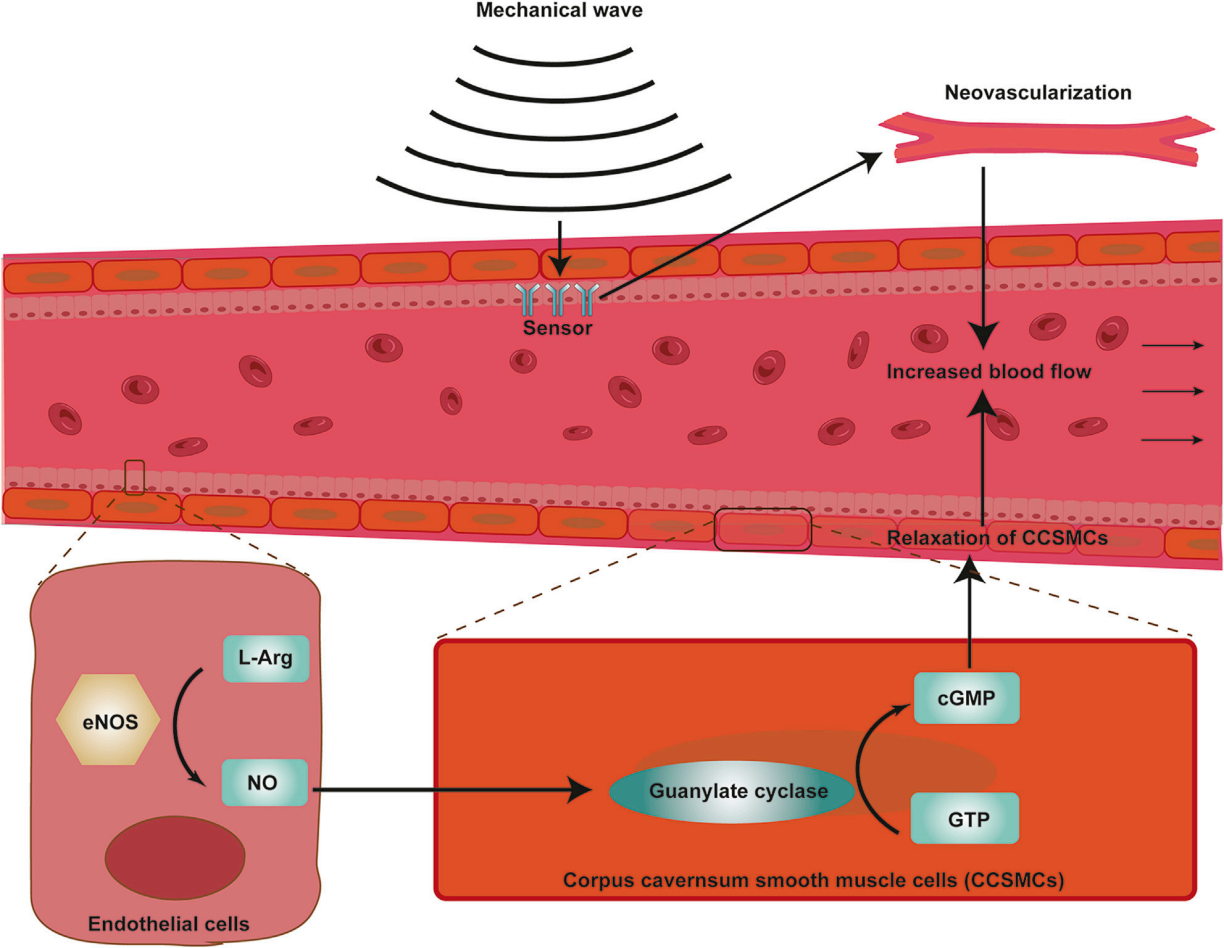
Mechanism of Li-ESWT for the treatment of ED. Mechanical waves stimulate the endothelial mechanosensors of the blood vessels, resulting in neovascularization. It also stimulates nNOS production in vascular endothelial cells, smooth muscle cells, and nerve cells, and cGMP production increases. Together, these promote increased blood flow to the penile corpus cavernosum and penile erection.

Electrical stimulation is one of the emerging technologies in clinical practice. It is a physical method that relies on the output of low-frequency pulsed current to treat diseases and is now widely used in urology, male surgery, gastrointestinal surgery, obstetrics, and gynecology, *etc.* (Qu et al., 2017; Wang et al., 2018b; Li et al., 2021). Especially in the field of treatment of ED, increasing numbers of studies have shown that the use of electrical stimulation techniques for ED can achieve positive outcomes (Figure 3).

According to some studies, the main principle of functional electrical stimulation for the treatment of ED include inducing penile endothelial proliferation and cavernous smooth muscle regeneration, promote NO release from cavernous endothelial cells (Gratzke et al., 2010), and producing cGMP, which can relax cavernous smooth muscle and raise cavernous body pressure (Hurt et al., 2002). According to the current state of clinical research, most studies are limited to the efficacy of functional electrical stimulation (FES) on ED. Some scholars believe that ES can stimulate peripheral nerve regeneration (Willand et al., 2016), which can dramatically improve the recovery of nerve function, and improve ED symptoms in turn. Regenerative electrical stimulation (RES) can treat ED patients with cavernous nerve damage through promoting nerve regeneration and restoring damaged nerve function (Balog et al., 2019). For example, in a study of a rat model of ED after nerve dissection, Shapira et al. found that short courses of electrical stimulation administered early in the course of nerve injury promoted recovery of nerve function (Shapira et al., 2019). Mendez et al. found that RES after facial nerve injury in rats accelerated facial nerve function and improved regeneration of facial nerve-specific pathways, and that ES significantly increased brain-derived neurotrophic factor (BDNF) expression in the nucleus of the cell body of motor neurons after injury, promoting repair of damaged nerve cells (Mendez et al., 2018). In summary, the main mechanism of RES is the upregulation of BDNF and its receptor, tyrosine kinase B (trkB), in motor neurons (Al-Majed et al., 2000; Balog et al., 2019), BDNF and the binding of trkB can promote nerve regeneration and the recovery of damaged nerve function (English et al., 2014).

Carboni et al. (Carboni et al., 2018) initially investigated the effects of FES on ED. They found that after 4 weeks of FES treatment, the patients’ IIEF-5 and Erection Hardness Score (EHS) significantly improved, indicating that FES had a positive therapeutic effect on ED. The study Shafik et al. also found that transcutaneous perineal ES can treat neurogenic ED (Shafik et al., 2008). Van et al. demonstrated that pelvic floor muscle function training combined with ES can produce positive results in the treatment of ED, with nearly half of the patients in the trial regaining normal erectile function (Van Kampen et al., 2003). Rislan et al. compared the therapeutic effects of ES and aerobic exercise on ED and found that ES was significantly more effective than aerobic exercise in the treatment of ED (Rislanu et al., 2020). Based on summarizing the existing relevant studies and experiments, due to the continuous progress of current electrical stimulation, ES for ED has increasingly obvious advantages, including: a. It is a simple and non-invasive physical therapy program (Lin and Chen, 2017); b. Clinically, ES treatment is much cheaper than other methods, and the patient compliance is relatively high (Azevedo Coste et al., 2017).

The advantages of low-frequency ES for ED include (Salonia et al., 2021): few side effects, non-invasive (Ismail and El-Sakka, 2016), easy operation; low cost, and a short single treatment time. The disadvantages include (Salonia et al., 2021): a lack of a systematic and standardized treatment plan, a lack of sufficient clinical case verification (Ismail and El-Sakka, 2016), the difficulty of applying individualized treatment for different causes. Therefore, the authors concluded that ES combined with akupunktur for ED could produce better results than ES treatment alone.

At present, akupunktur combined with electrophysiological technology is relatively mature. The update of equipment needs to closely match the development of electrophysiological technology, and ES treatment also requires more advanced techniques and equipment. It can be combined with drugs, multi-mechanism, and multi-target therapy. It can be used to treat erectile dysfunction by electrically stimulating the acupoints.

### 4.3 Low-intensity extracorporeal shock wave therapy (Li-ESWT)

**FIGURE 4:**
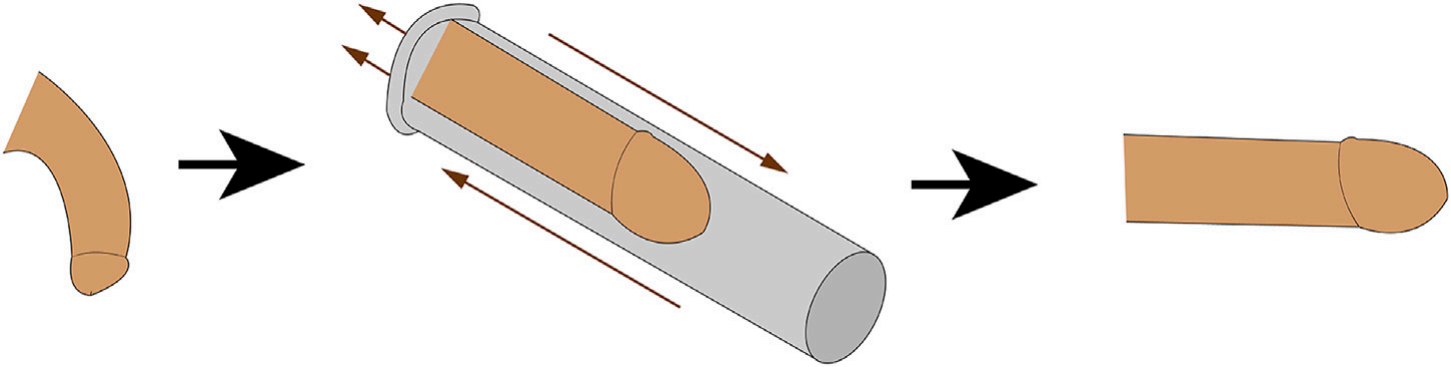
Mechanism of VED for the treatment of ED. Negative pressure dilates the cavernous sinuses, increasing their arterial and venous blood perfusion and ultimately achieving penile erection.

Since its initial introduction in 2010, Li-ESWT has gained popularity as a treatment for ED (Vardi et al., 2010) (Figure 4). Li-ESWT can achieve the purpose of treating ED primarily by stimulating tissue repair and vascular regeneration (Stoykov et al., 2020). Li-ESWT was initially introduced as urinary system lithotripsy (Wang and Zhou, 2015), however, many studies reported the benefits of Li- ESWT in different medical fields such as musculoskeletal diseases, wound, and bone healing disorders, ischemic heart disease, and spastic tension (Dumfarth et al., 2008; Gadomski et al., 2018; Yue et al., 2021). At present, Li-ESWT has been widely used in the clinical treatment of ED.

The mechanism of Li-ESWT treatment for ED is still unknown. Its effects could be caused by the induction of mechanical stress, which could result in neovescularization, the recruitment of stem cells and growth factors, an improvement in blood flow, and nerve regeneration (Gruenwald et al., 2013; Mason et al., 2022). Some studies have shown that Li- ESWT can promote the expression of neuronal nitric oxide synthase (nNOS) in endothelial cells, smooth muscle cells, and nerve cells (Yao et al., 2022). However, according to some studies, Li-ESWT does not rely on nNOS and guanosine cyclic phosphate to improve erectile function (Assaly-Kaddoum et al., 2016). Up to now, no study has confirmed the specific mechanism of Li-ESWT in the treatment of ED.

Oginski n et al. Carried out a study in which 50 ED patients were treated with Li-ESWT once a week for 6 weeks. It was considered a successful treatment if the IIEF-5 score increased by ≥ 5 points or the erectile stiffness score increased by ≥ 3 points. Among them, 56% of patients were shown to be treated effectively; 50% of patients were improved in the first 3 months, and in which 16% continued for 6 months. Another 3 cases had improved erectile function 6 months after the treatment. In addition, the effect was significantly improved for patients with cardiovascular risk factors (*p* = .026) (Oginski et al., 2022). This study proves that Li-ESWT is an effective but short-term treatment method for ED patients, especially those with cardiovascular diseases. Liu et al. (Liu et al., 2022) conducted a meta-analysis to assess the efficacy of Li-ESWT for ED and found that Li-ESWT significantly improves erectile function in patients with mild and moderate ED. Moreover, its safety is very high, and so far there are few reports of adverse effects (Pai et al., 2021). There is increasing evidence that Li-ESWT causes minor damage to vital organs such as the heart while improving myocardium, bladder, joints, and penis function (Jiang et al., 2021).

A study by Zewin et al. investigated the role of Li-ESWT in penile rehabilitation after nerve-preserving radical prostatectomy in men. The Li-ESWT treatment group in the study showed a significant increase in total IIEF score, sexual satisfaction, overall satisfaction domain score, and EHS score throughout the follow-up period, demonstrating its clinical therapeutic properties (Zewin et al., 2018). Moreover, in a study of 350 ED patients by Leonid Spivak et al., Li-ESWT improved phosphodiesterase five inhibitor sensitivity in 55% of patients who did not respond to phosphodiesterase five inhibitors (Spivak et al., 2021). Li-ESWT is relatively safe for short-term treatment in multiple studies, but there is a lack of long-term studies confirming the safety of Li-ESWT. With the shortcoming of expensive, Li-ESWT has not been approved by the FDA for the treatment of ED.

### 4.4 Akupunktur treatment

ED is called impotence in Traditional Chinese medical (TCM). Some TCM methods are frequently used to treat ED, and the most commonly used method is akupunktur (Tan et al., 2021). Akupunktur is the umbrella term for acupuncture and moxibustion. Acupuncture is the procedure of inserting filiform needles into specific points on the patient’s body, known as acupoints, and using acupuncture techniques such as twisting and lifting to treat disease (Zhou et al., 2021). Moxibustion is the practice of smoked burning the skin with burning Ai velvet according to certain acupoints and using the heat to stimulate the treatment of diseases. TCM believes that these acupoints can control and regulate the flow of qi, as well as its distribution and excretion in the viscera, in order to maintain the balance between the internal and external environments (Zhou et al., 2021). It has been reported that akupunktur can control the release of nitric oxide and some neuropeptides involved in the erectile process (Wang et al., 2022b). Acupuncture can also improve blood circulation and regulate the sensitivity of nerves to relieve the symptoms of ED patients (Wang et al., 2019b). However, the available evidence is still insufficient to demonstrate that akupunktur is an effective method for ED. Therefore, the therapeutic effect of akupunktur on ED requires further investigation (Li et al., 2017).

### 4.5 Discussion

Overall, the current treatment for ED includes medication, physical therapy, psychotherapy, and surgery. Medication, especially PDE-5 inhibitors, is still the first-line treatment (Yuan et al., 2021), among which sildenafil, vardenafil, tadalafil, and avanafil have better efficacy (Liu et al., 2020a). Although the results are encouraging, many patients do not respond to these medications, are unable to tolerate the side effects, or relapse after discontinuation. Therefore, it is critical to research physical therapy methods and mechanisms related to ED. It could also be used as a springboard for the development of more clinically relevant physical therapy approaches for the treatment of ED. However, each method has its advantages and limitations. At present, the cost of various physical therapies varies in clinical practice. ES, VED, and acupuncture are relatively inexpensive for most patients, while Li-ESWT treatment is relatively expensive. When faced with patients with ED in clinical settings, physicians should establish a treatment plan that considers the severity of the patient’s ED and the patient’s financial situation. It is possible to start with a relatively inexpensive treatment modality, alone or in combination, such as ES or a combination of ES and VED. When the program does not work, then switch to other modalities.

As the prevalence of ED increases, there is a greater incentive to find safer, more effective, and simpler treatments. Pharmacological and physical therapies paid more attention on symptoms control but not the function restore. More attention has recently focused on the latest technologies of gene therapy and stem cell transplantation. Bone marrow-derived and adipocyte-derived stem cells have been used in animal models with dramatic results. We are looking forward that these treatments could help a large number of ED patients to regain their strength in the coming years.

## 5 Conclusion

We have now reviewed literatures about the pathogenesis of ED or some of the factors that influence the development of the disease. In this review, the authors detailed the various etiologies of ED and the pathogenesis of each etiology, and presented the various common clinical diagnostic and physical therapy devices currently available for ED and the advantages and disadvantages of each device. This will enable clinicians to provide individualized treatment plans for each patient based on the different etiopathogenic factors, and to improve the condition of ED patients in the future.

## Glossary

ED: erectile dysfunction
NO: nitric oxide
cGMP: cycloguanosine monophosphate
RhoA: ras homolog gene family
AGEs: advanced glycation end products
PKG-1: cGMP dependent protein kinase-1
NPT: nocturnal penile tumescence
PDEi-5: phosphodiesterase inhibitor-5
PPDU: pharmaco penile duplex ultrasonography
PGE1: prostaglandin 1
PSV: peak systolic velocity
EDV: end-diastolic velocity
IPA: internal pudendal arteriography
DSA: digital subtraction angiography
D-e CTA: dual-energy CT arteriography
4D-CTA: 320-Detector row dynamic volume CT
VED: vacuum erectile device
ES: electrical stimulation
FES: functional electrical stimulation
RES: regenerative electrical stimulation
BDNF: brain-derived neurotrophic factor
trkB: tyrosine kinase B
EHS: erection hardness score
Li-ESWT: low-intensity extracorporeal shock wave therapy
nNOS: neuronal nitric oxide synthase
TCM: traditional chinese medical
